# Antinociceptive and Anti-Inflammatory Effects of Octacosanol from the Leaves of *Sabicea grisea* var. *grisea* in Mice

**DOI:** 10.3390/ijms13021598

**Published:** 2012-02-02

**Authors:** Anderson Marques de Oliveira, Lucia M. Conserva, Jamylle N. de Souza Ferro, Fabíola de Almeida Brito, Rosângela P. Lyra Lemos, Emiliano Barreto

**Affiliations:** 1 Instituto de Química e Biotecnologia, Universidade Federal de Alagoas, Maceió/AL 57072-970, Brazil; E-Mails: andermarx@yahoo.com.br (A.M.O.); lmc@qui.ufal.br (L.M.C.); 2 Laboratório de Biologia Celular, Universidade Federal de Alagoas, Maceió/AL 57072-970, Brazil; E-Mails: mylla_champras@hotmail.com (J.N.S.F.); fabiolabrito@hotmail.com (F.A.B.); 3 Instituto do Meio Ambiente do Estado de Alagoas, Maceió/AL 57017-320, Brazil; E-Mail: rosalyralemos@gmail.com

**Keywords:** octacosanol, anti-inflammatory, antinociception, TNF-α, *Sabicea grisea*

## Abstract

*Sabicea* species are used in the Amazon for treatment of fever and malaria, which suggests that its chemical constituents may have some effect on pain and inflammation. Phytochemical analysis of the hexane fraction obtained from the crude ethanol extract from *Sabicea grisea* var. *grisea* Cham. & Schltdl (Rubiaceae), an endemic plant in Brazil, resulted in the isolation of octacosanol. This study investigated the antinociceptive and anti-inflammatory effects of the octacosanol in different experimental models. The crude ethanolic extract and hexane fraction obtained from the leaves of *S. grisea* produced an inhibition of acetic acid-induced pain. Moreover, octacosanol isolated from the hexane fraction produced a significant inhibition of pain response elicited by acetic acid. Pre-treatment with yohimbine, an alpha 2-adrenergic receptor antagonist, notably reversed the antinociceptive activity induced by octacosanol in the abdominal constriction test. Furthermore, mice treated with octacosanol did not exhibit any behavioral alteration during the hot plate and rota-rod tests, indicating non-participation of the supraspinal components in the modulation of pain by octacosanol with no motor abnormality. In the formalin test, octacosanol did not inhibit the licking time in first phase (neurogenic pain), but significantly inhibited the licking time in second phase (inflammatory pain) of mice. The anti-inflammatory effect of octacosanol was evaluated using carrageenan-induced pleurisy. The octacosanol significantly reduced the total leukocyte count and neutrophils influx, as well as TNF-α levels in the carrageenan-induced pleurisy. This study revealed that the mechanism responsible for the antinociceptive and anti-inflammatory effects of the octacosanol appears to be partly associated with an inhibition of alpha 2-adrenergic transmission and an inhibition of pathways dependent on pro-inflammatory cytokines. Finally, these results demonstrated that the octacosanol from the leaves of *S. grisea* possesses antinociceptive and anti-inflammatory activities, which could be of relevance for the pharmacological control of pain and inflammatory processes.

## 1. Introduction

Octacosanol, a long-chain aliphatic alcohol, found abundantly in wheat germ oil or rice bran oil and in many other plants [1], presents a variety of important biological activities, including antioxidant and ergogenic properties and anti-parkinsonism [2,3]. In addition, this alcohol and its synthetic analogue, octacosa-10,19-dien-1-butanol, both presented an antiangiogenic effect and an inhibition of the activity of matrix metalloproteinase [4,5]. Mixtures of long-chain aliphatic alcohols containing octacosanol (policosanol) have been shown to decrease the weight of adipose tissue [6] and to inhibit cholesterol biosynthesis [7]. Furthermore, studies have shown that policosanol monotherapy reduces LDL-cholesterol and increases HDL-cholesterol [8]. Besides these effects on lipid metabolism, policosanol also presents a wide range of pharmacological activities, such as reducing platelet aggregation [9], as antiulcer [10] and as anti-inflammatory [11,12].

Identification of bioactive compounds from plants has become a highly active area of pharmaceutical research. Such compounds have been applied in the treatment of different conditions, including anxiety, pain, and inflammation [13–15]. Thus, the evaluation of pharmacological effects on these conditions can be used as a strategy for discovering new drugs of plant origin.

Rubiaceae family is considered one of the biggest families among the Angiosperms, comprising around 637 genera and approximately 10,700 species, which are of great importance to the food, ornamental and pharmaceutical industries [16]. Rubiaceae species are known to be used as bioproducers of alkaloids, tannins, saponins, steroids, fatty acids, fatty alcohols, terpenes and flavonoids, besides reports that some species are important to traditional medicine [17–19]. Considering the wealth of metabolites of the family Rubiaceae, the phytochemical study of species that represent the genera of this family, especially of the genus *Sabicea* whose chemistry is still little known, leads are expected in the discovery of naturally active substances although as yet they are little explored.

The genus *Sabicea* is the most species-rich of the tribe Sabiceae with 146 species of shrubs and woody climbers distributed in mainland Africa and the Neotropics [20]. Species of this genus are used in folk medicine for treating epilepsy [13], fever, vomiting, insomnia [21], stomach ache, dysentery and malaria [22]. In the Amazon, the use for the treatment of fever and malaria suggests that chemical constituents present in this genus may have some effect on pain and inflammation. *Sabicea grisea* Cham. & Schltdl. var. *grisea* is endemic in Brazil and until now, no phytochemical or biological study has been performed with this species. Based on these observations, the present study used different experimental models in mice to test the hypothesis that octacosanol isolated from the leaves of *S. grisea* could demonstrate properties antinociceptive and anti-inflammatory.

## 2. Results and Discussion

### 2.1. Extraction and Isolation of Octacosanol

**Figure 1:**
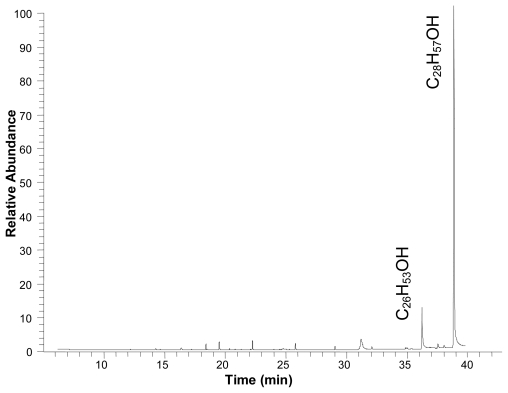
Gas chromatogram profile of octacosanol isolated from the leaves of *S. grisea*.

Powder of the dried leaves of *S. grisea* was extracted at room temperature with 90% ethanol. The obtained crude ethanol extract was partitioned between hexane, chloroform and hydro-alcoholic solution (7:3). The hexane fraction was further fractionated on a silica gel column. Fractions with similar thin layer chromatography profiles were pooled. The remaining fractions were subjected to washings with hexane that resulted in the isolation of octacosanol (C_28_H_57_OH). Verification of the purity of octacosanol was carried out by gas chromatography. The GC data revealed that a major peak was eluted at 38.79 min. However, minor impurities (especially hexacosanol at 36.08 min—C_26_H_53_OH) were also detected as per the GC profile indicating that the compound was about 90% pure (Figure 1).

### 2.2. Writhing Response Induced by Acetic Acid in Mice

**Figure 2:**
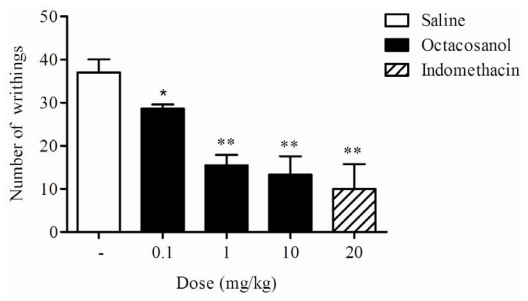
Effect of the octacosanol on acetic acid-induced writhing response in mice. The values are expressed as the mean ± S.E.M. of six mice. * *P* < 0.05 and ** *P* < 0.01 indicate statistically significant differences from the saline-treated group.

The writhing test has long been used as a screening tool for the assessment of analgesic properties of new substances [23]. It is accepted that acetic acid acts by releasing endogenous substances, such as prostaglandins and cytokines, which excite primary sensor neurons [24]. In this study, the crude ethanol extract and its sequential fraction in hexane from *S. grisea* at 100 mg/kg (i.p.) significantly reduced the number of writhes (to 8.25 ± 2.65 and 9.50 ± 5.70 abdominal writhes), respectively, in relation to the control group (37.00 ± 3.74 abdominal writhes). Administration of octacosanol (0.1, 1 and 10 mg/kg, i.p.), 60 min before the acid injection, produced a significant inhibition of abdominal constrictions in mice (Figure 2). The percentage of inhibition produced by octacosanol at all three doses tested was 22.5, 58.1, and 63.9%, respectively, compared to the control. This was also comparable with indomethacin (20 mg/kg, i.p.), a standard nonsteroidal anti-inflammatory drug used as positive control, that also produced a significant inhibition (72.9%). In fact, acetic acid causes an increase in peritoneal fluid levels of prostaglandin E2 (PGE2) and prostaglandin F2 (PGF2), in part involving peritoneal receptors and causing inflammatory pain by inducing capillary permeability [25]. Thus, our results suggest that the action of octacosanol may be linked partly to the inhibition of cyclooxygenase in peripheral tissues, thereby reducing PG synthesis and interfering with the mechanism of transduction in primary afferent nociceptors. In addition, this data suggests octacosanol is one of the main active compounds present in the leaves of *S. grisea*.

**Figure 3:**
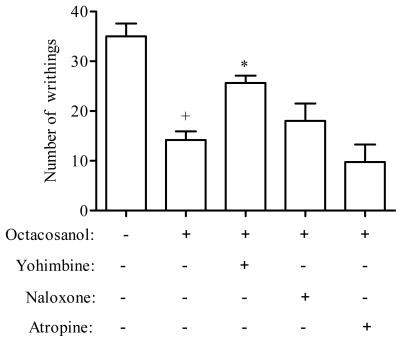
Effect of pretreatment of animals with yohimbine (2 mg/kg, s.c.), naloxone (5 mg/kg, s.c.) and atropine (2 mg/kg, s.c.) on the antinociceptive profile of the octacosanol against acetic acid-induced writhing in mice. ^+^ *P* < 0.001, indicate statistically significant differences compared to untreated group. * *P* < 0.05, indicate statistically significant differences compared to octacosanol-treated group. Under the graph, the signs + and − indicate the presence or absence of the respective treatment.

To assess the possible mechanisms of antinociceptive activity of the octacosanol, we examined the effects of naloxone, yohimbine and atropine on the writhing test induced by acetic acid. The results presented in Figure 3 show that pre-treatment of the mice with yohimbine (1 mg/kg), an alpha 2-adrenergic receptor antagonist, was able to partially revert the antinociceptive effect of octacosanol (10 mg/kg, i.p.), suggesting a possible involvement of alpha 2-adrenergic receptors in the actions of octacosanol.

The opiate mechanism was analyzed by testing the effect of naloxone on the octacosanol-induced antinociception. The dose of naloxone used in the experiments was high enough to block opiate receptors [26]. In the present study, naloxone was completely inactive as antagonist, thus precluding involvement of the central opiate mechanism in the antinociception of octacosanol (Figure 3). Similarly, a muscarinic-mediated mechanism can also be excluded due to the lack of antagonism by atropine (Figure 3).

In fact, the roles of opioid, muscarinic and adrenergic receptors in the regulation of the modulation of nociceptive processing have been demonstrated in several previous studies [27–29]. Here, we observed that alpha 2-adrenergic receptors, but not opioid and muscarinic receptors, appear to be involved in octacosanol-induced antinociception.

### 2.3. Effect on Rota-Rod Assay in Mice

The writhing test shows good sensitivity, as it allows for the effects of weak analgesics, but it does show poor specificity because the abdominal writhing response may be suppressed by muscle relaxants and other drugs, leaving scope for the misinterpretation of results [30]. This can be avoided by complementing the test with other models of nociception and a motility test. For this reason, octacosanol was examined for its inhibitory action on motility in the rota-rod test. The rota-rod procedure was described by Dunham and Miya [31] and is suitable for detecting motor impairment due to pharmacological agents such as skeletal muscle relaxants or central nervous system depressants. In this study, octacosanol-treated mice did not show any significant motor performance alterations with the dose of 10 mg/kg i.p. (232.02 ± 39.00 s) as compared to saline-treated animals (205.33 ± 37.97 s) in the rota-rod test. As expected, diazepam (positive control, 10 mg/kg, i.p.), the most widely used benzodiazepine derivative, that causes sedation and inhibits locomotor activity, reduced the motor performance time of mice after 30 min of treatment (66.25 ± 15.39 s). Thus, as octacosanol did not affect the motor performance of mice in the rota-rod test, the effects of relaxing or motor deficits could be excluded.

### 2.4. Effect on Hot-Plate Latency Assay in Mice

It has been well documented that the hot plate is a central antinociceptive test in which opioid agents exert their analgesic effects via supraspinal and spinal receptors [30]. In this assay, octacosanol (1 and 10 mg/kg, i.p.) did not exert any significant changes in the response of latency (9.41 ± 2.29 and 10.08 ± 1.15 s, respectively) against the thermal stimulus-induced nociception, as compared to control (7.03 ± 0.88 s), while morphine (positive control; 5 mg/kg, i.p.) significantly increased the latency response (18.99 ± 1.41 s). The treatment with octacosanol was found to induce an antinociceptive response in the abdominal writhing test. This treatment was ineffective on the hot plate test which indicates a non-participation on thermal stimulation associated with central neurotransmission whereby heat activates nociceptors by driving the momentum of the dorsal horn of the spinal cord which subsequently affects cortical centers.

### 2.5. Effects on Formalin-Induced Nociception in Mice

The formalin-induced paw licking test is commonly employed as a model of acute tonic pain, characterized by the presence of a distinct biphasic nociceptive response. The first phase corresponds to neurogenic pain as it involves direct activation of formalin on transient receptor, sensory C-fibers, that reflect centrally mediated pain [32]. The second phase of the nociceptiva reaction, also known as inflammatory pain, is mediated by a combination of peripheral input from inflammatory mediators released from injured tissues, which result in the sensitization of central nociceptive neurons [33]. It was reported that substance P and bradykinin participate in the appearance of the first-phase responses, while histamine, serotonin, prostaglandin and bradykinin are involved in the second-phase responses. It has been well established that drugs that act primarily on the central nervous system inhibit both phases equally, while peripherally acting drugs inhibit only the second phase [34].

**Figure 4:**
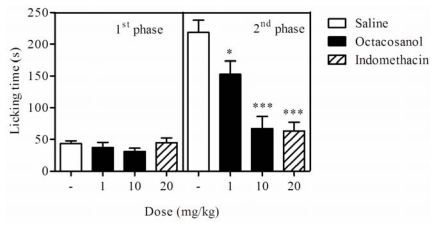
Effect of octacosanol (1 and 10 mg/kg) and indomethacin (positive control; 20 mg/kg, i.p.) on nociception induced by formalin during the first 5 min (1^st^ phase), and during 15–30 min (2^nd^ phase). Data are expressed as mean ± SEM (*n* = 6). * *P* < 0.05 and *** *P* < 0.001 compared to respective saline-treated group.

In the present study, the licking time for the first phase was 44.00 ± 6.00 s and for the second phase 218.66 ± 23.73 s in control groups (vehicle). Using the two higher doses (1 and 10 mg/kg) able to inhibit nociceptive stimulus induced by acetic acid, the pretreatment of animals for 60 min with octacosanol had no significant effect on the duration of licking activity in first phase (37.20 ± 8.98 s and 31.00 ± 6.36 s, respectively), whereas both doses produced a marked reduction of the duration of the licking in the second phase (152.75 ± 23.97 s and 67.25 ± 21.97 s, respectively). Indomethacin (20 mg/kg), a reference drug, significantly inhibited the pain only in the second phase of the test (63.40 ± 15.35 s). Thus, our results showed that octacosanol suppressed the nociceptive response in the inflammatory phase but not in the neurogenic phase of the formalin test (Figure 4).

### 2.6. Effects on Carrageenan-Induced Pleurisy in Mice

Pleurisy induced by phlogistic agents is a widely accepted model for the evaluation of the anti-inflammatory effect of compounds obtained from plants [35]. To assess the anti-inflammatory activity, octacosanol was evaluated by the carrageenan-induced pleurisy model. Carrageenan-induced pleurisy is a classical model of acute inflammation involving various types of chemical mediators of inflammation such as vasoactive amines, complement fragments, prostaglandins, and cytokines [36]. The administration of carrageenan into the pleural space is characterized by protein-rich fluid accumulation and polymorphonuclear leukocytes infiltration, which are important processes of inflammatory pathology [35].

As shown in Table 1, substantially fewer leukocytes were observed in animals injected with vehicle. Administration of carrageenan into the pleural space of mice induced an increase in total leukocyte count, characterized by an increase in the number of neutrophil and mononuclear cells 4 h after injection. Treatment with octacosanol, administrated 1 h before carrageenan, at doses of 1 and 10 mg/kg i.p., was able to suppress significantly the recruitment of total leukocytes to the mouse pleural cavity.

Others studies have demonstrated that mixtures of aliphatic alcohols are effective in preventing the neutrophil infiltration in the inflamed tissue [12]. In fact, Carbajal *et al.* [37] reported that a natural mixture of high molecular weight alcohols shows an anti-inflammatory activity which is effective in both chronic and acute inflammation. Here, our study demonstrated that octacosanol was able to inhibit the neutrophils influx in mice, but did not alter mononuclear cells migration in the carrageenan-induced pleurisy.

The intrapleural administration of carrageenan into the pleural space induces non-specific inflammatory reactions, which induce the activation of innate immunity and the peripheral release of tumor necrosis factor-alpha (TNF-α) [38]. The TNF-α has been shown to have many biological activities in the inflammatory reaction, including fever, accumulation of neutrophils in local tissues, induction of vascular adhesion molecules and stimulation of acute phase protein synthesis [39]. Our results show that 4 h after the injection of carrageenan a substantial increase in the amount of TNF-α was found in pleural exudates when compared with saline injected mice (Table 2). A significant reduction in TNF-α concentration was observed in the treated group with octacosanol (1 and 10 mg/kg, i.p.) and indomethacin (20 mg/kg, i.p.) as compared to the carrageenan-treated group.

Many studies have shown that fatty alcohols also reduce the TNF-α production in both *in vitro* and *in vivo* [11,40]. Together, our results show that the octacosanol inhibited the influx of total leukocytes into the pleural cavity triggered by carrageenan, due to the inhibition of neutrophils recruitment. In addition, this compound showed the ability to decrease TNF-α in the pleural fluid of mice with carrageenan-induced inflammation.

## 3. Experimental Section

### 3.1. Plant Material

Leaves of *Sabicea grisea* Cham. & Schltdl. var. *grisea* were collected in May 2008, at the Horto Municipal de Maceió, Alagoas State, Brazil, and identified by Rosangela P. L. Lemos of the Instituto do Meio Ambiente do Estado de Alagoas, where a voucher specimen was deposited (MAC-11356).

### 3.2. Preparation of Extracts, Purification and Structure Elucidation of Octacosanol

Powder of the dried leaves of *S. grisea* was extracted at room temperature with 90% ethanol. The solution was filtered using a Whatman N° 1 filter paper under suction and concentrated to dryness at 50 °C under reduced pressure. The obtained crude ethanol extract was partitioned between hexane, chloroform and hydro-alcoholic solution (7:3). The hexane fraction was further fractionated on a silica gel column using hexane, containing increasing amounts of ethyl acetate. The remaining fractions were subjected to washings with hexane that resulted in the isolation of octacosanol (45 mg), m.p. 81–82 °C. The concentration of octacosanol was 88.23 μg per 1 g dry weight of leaves. This compound was structurally characterized based on its GC-MS, and ^1^H and ^13^C NMR analyses together with available literature [41,42]. GC-MS analysis of octacosanol was carried out with instrument GC–MS system (Varian 3200 and Finnigam Incos-XL) under the following conditions: DB-5 fused silica column (30 m length, 0.25 mm i.d., 0.25 mm film thickness); injector temperature, 220 °C; temperature programmed at 60–270 °C at 3 °C/min; injection type, splitless (1 μL of a 1:1000 *n*-hexane solution); carrier gas, helium, adjusted to a linear velocity of 32 cm/s (measured at 100 °C); mass spectra, 70 eV, in EI mode; ion source temperature, 180 °C; scan mass range, 25–700 u.

Immediately prior to use, the crude ethanolic extract, hexane fraction and octacosanol were dissolved in 200 μL of absolute ethanol and 3800 μL of saline (NaCl, 0.9%) to make a concentration of 2 mg/mL, which was diluted to achieve the required concentrations for subsequent experiments.

### 3.3. Animals

Male Swiss mice weighing 18–22 g were provided by the Central Biotery of Universidade Federal de Alagoas (UFAL) breeding unit. The animals were maintained with free access to food and water and kept at 22 ± 2 °C with a controlled 12 h light-dark cycle at Instituto de Ciências Biológicas e da Saúde. Experiments were performed during the light phase of the cycle. The animals were allowed to adapt to the laboratory for at least 2 h before testing and used only once. Animal care and the experimental protocol followed the principles and guidelines outlined by the Brazilian College of Animal Experimentation (COBEA) and were approved by the local ethical committee (License no. 23065.009301/2009-96).

### 3.4. Acetic Acid-Induced Writhing Response in Mice

Antinociceptive activity was evaluated using the test of abdominal writhing induced by acetic acid in mice [23]. Abdominal writhing in mice was caused by the intraperitoneal (i.p.) injection of 0.6% acetic acid, 0.1 mL/10 g body weight. Animals were divided into groups of six mice. Control animals received an i.p. injection of the same volume of vehicle (saline, NaCl 0.9%). One group of mice received indomethacin (20 mg/kg, i.p.) as a reference compound, and the other three groups received different doses of octacosanol (0.1, 1 and 10 mg/kg body weight, i.p.) 60 min before stimulation with acetic acid. The doses of octacosanol used in this study were obtained from previous reports [3,43]. Solutions containing octacosanol were placed on ultrasound for 5 min, before using. Five minutes after the acetic acid injection, the number of times that each animal presented abdominal constriction was counted for 10 consecutive min. The percentage of inhibition was determined for each experimental group using the following formula [14]:

Inhibition %=100-T×100/C(%) or T×100/C-100(%)

Where *C* and *T* indicate non-treated (vehicle) and drug-treated, respectively.

### 3.5. Analysis of Possible Antinociceptive Mechanisms of Octacosanol

To assess the possible mechanisms involved in the antinociceptive action of octacosanol, mice were previously treated with different antagonists in the acetic acid model. To determine the possible participation of different systems, the following antagonists were used; naloxone (5 mg/kg), atropine (5 mg/kg), and yohimbine (1 mg/kg), administered subcutaneous (s.c.) 15 min before the octacosanol (10 mg/kg, i.p.). All the reagents were obtained from Sigma.

### 3.6. Hot-Plate Latency Assay in Mice

The hot-plate test was used to measure reaction times according to the method described by Eddy and Leimback (1953) [44]. Animals were placed individually on a hot plate metallic surface (Insight^®^, Brazil; model EFF-361) maintained at 54 ± 1 °C, and the time between placement of the animal on the hot plate and the occurrence of either licking of the hind paws, shaking, or jumping off the surface was recorded as reaction time (s). Reaction time was measured at 60 min following treatment with saline (i.p.), octacosanol (1 and 10 mg/kg, i.p.) or morphine (5 mg/kg, i.p.), with a cut-off time of 20 s. Each experimental group contained at least six animals.

### 3.7. Formalin-Induced Nociception in Mice

Mice were injected with formalin (2.5%, 20 μL) in the subplantar area of the right hind paw. The time of paw licking (in seconds) was determined over 0–5 min (first phase—neurogenic) and 15–30 min (second phase—inflammatory) after formalin injection [34]. Animals (*n* = 6) were pretreated with octacosanol (1 and 10 mg/kg, i.p.) 60 min before intraplantar injection of the stimulus or the reference compound, indomethacin (20 mg/kg, i.p.), 1 h before administration of formalin. Control animals were treated with sterile saline.

### 3.8. Rota-Rod Assay

The motor coordination of the animals was evaluated in a rota-rod apparatus (Insight^®^, Brazil; model EFF-412). The animals were trained on the apparatus for 2 days before the experiment. On the day of the experiment, mice were treated with octacosanol (10 mg/kg, i.p.) or diazepam (10 mg/kg, i.p) and tested in the rota-rod from 60 min after their administration [31]. After treatment, the motor performance time (s) was recorded with a stopwatch for up to 240 s.

### 3.9. Carrageenan-Induced Pleurisy in Mice

Pleurisy was induced in mice by intrapleural (i.pl.) administration of 100 μL of 1% (w/v) carrageenan suspension in saline solution. A specially adapted 13 × 5 needle was introduced into the right side of the thoracic cavity of mice to inject the carrageenan solution, and an equal volume of sterile saline was injected into the controls. After 4 h of the carrageenan suspension, the animals were sacrificed in a CO_2_ gas chamber and the thoracic cavity was opened and washed with 1 mL of PBS containing EDTA (10 mM). Pleural wash samples were diluted in Turk fluid (2% acetic acid) for total leukocyte counts using Neubauer chambers. Cytospin preparations of the samples were stained with May–Grunwald–Giemsa for the differential leukocyte count, which was performed under the immersion objective of a light microscope. The animals (six in each group) were pre-treated via the intraperitoneal (i.p.) route with octacosanol (1 and 10 mg/kg, i.p.) 60 min prior to the induction of pleurisy. Thus, parallel groups of animals were pre-treated (60 min before pleurisy induction) with indomethacin (20 mg/kg, i.p.) and 4 h later the same inflammatory parameters were evaluated.

### 3.10. Quantification of TNF-α in Carrageenan-Induced Pleurisy

Levels of tumor necrosis factor-alpha (TNF-α) in the pleural fluid of the animals treated with octacosanol (1 and 10 mg/kg, i.p.) and indomethacin (20 mg/kg, i.p.) were evaluated by sandwich ELISA, using mouse TNF-α ELISA Ready-SET-Go reagent set (eBioscience) (San Diego, CA, USA), according to the manufacturer’s instructions.

### 3.11. Statistical Analysis

Data are reported as mean ± standard error of the mean (SEM) and were analyzed with the use of GraphPad Prism^®^ software, version 5.0 (San Diego, CA, USA). Statistical differences between groups were determined when necessary using Student’s *t* test or via two-way analysis of variance followed by Student-Neuman-Keuls test. P values less than 0.05 were considered statistically significant.

## 4. Conclusions

The present study demonstrated, for the first time, that octacosanol, a long-chain aliphatic alcohol isolated from the leaves of *Sabicea grisea* var. *grisea*, possesses a peripheral antinociceptive effect that is probably mediated by the alpha 2-adrenergic receptors. Furthermore, this compound inhibited both the influx leuckocyte as well as levels of TNF-α in the carrageenan-induced pleurisy model. Based on these findings, octacosanol may be useful in developing new strategies for preventing pain and inflammation.

## Figures and Tables

**Table 1:** Effect of octacosanol on total leukocyte, neutrophil and mononuclear cells induced by carrageenan in the pleural cavity of mice.

Groups of animals	Total leukocytes (×10^6^ cells/cavity)	Neutrophils (×10^6^ cells/cavity)	Mononuclear cells (×10^6^ cells/cavity)
Basal	1.86 ± 0.14	0.12 ± 0.13	1.74 ± 0.16
Cg	11.85 ± 2.19 +++	8.05 ± 1.34 +++	3.22 ± 0.39 +
Cg + Octacosanol (1 mg/kg)	5.70 ± 1.34 **	2.74 ± 0.53 ***	2.89 ± 0.34
Cg + Octacosanol (10 mg/kg)	4.68 ± 0.51 ***	2.22 ± 0.22 ***	2.45 ± 0.36
Cg + Indomethacin (20 mg/kg)	3.26 ± 0.48 ***	1.24 ± 0.18 ***	1.83 ± 0.36

Each value represents the mean ± S.E.M. of six animals;

Basal = animals that received injection of vehicle in the cavity; Cg = carrageenan;

+ *P* < 0.05 and

+++ *P* < 0.001 as compared to saline-stimulated group;

** *P* < 0.01 and

*** *P* < 0.001 as compared to untreated (saline) stimulated group.

**Table 2:** Effect of octacosanol on tumor necrosis factor-alpha (TFNα) levels in the pleurisy induced by carrageenan in mice.

Groups of animals	TNF-α (pg/mL)	Inhibition (%)
Basal	19.53 ± 11.49	-
Cg	164.50 ± 50.99 ++	-
Cg + Octacosanol (1 mg/kg)	22.56 ± 14.80 **	86.28
Cg + Octacosanol (10 mg/kg)	24.17 ± 12.50 **	85.30
Cg + Indomethacin (20 mg/kg)	19.15 ± 4.43 **	88.35

Each data represents the mean ± S.E.M. of six mice;

Basal = animals that received injection of vehicle in the cavity; Cg = carrageenan;

++ *P* < 0.01 as compared to saline-stimulated group;

** *P* < 0.01 as compared to untreated (saline) stimulated group.
